# Need and Seek for Dietary Micronutrients: Endogenous Regulation, External Signalling and Food Sources of Carotenoids in New World Vultures

**DOI:** 10.1371/journal.pone.0065562

**Published:** 2013-06-13

**Authors:** Guillermo Blanco, Dámaso Hornero-Méndez, Sergio A. Lambertucci, Luis M. Bautista, Guillermo Wiemeyer, José A. Sanchez-Zapata, Juan Garrido-Fernández, Fernando Hiraldo, José A. Donázar

**Affiliations:** 1 Department of Evolutionary Ecology, National Museum of Natural History (CSIC), José Gutiérrez Abascal 2, Madrid, Spain; 2 Departamento de Biotecnología de Alimentos, Instituto de la Grasa (CSIC), Avda. Padre García Tejero, Seville, Spain; 3 Laboratorio Ecotono-INIBIOMA (CONICET-Universidad Nacional del Comahue), Quintral 1250, Bariloche, Río Negro, Argentina; 4 Jardín Zoológico de la Ciudad de Buenos Aires, Rep. de la India 3000 (CP 1425), Buenos Aires, Argentina; 5 Department of Applied Biology, University Miguel Hernández, Ctra. Beniel Km 3.2, Orihuela, Alicante, Spain; 6 Department of Conservation Biology, Estación Biológica de Doñana (CSIC), c/Americo Vespucio s/n, Seville, Spain; Arizona State University, United States of America

## Abstract

Among birds, vultures show low concentrations of plasma carotenoids due to the combination of their large size, general dull colouration and a diet based on carrion. We recorded the concentration of each carotenoid type present in plasma of the Andean condor (*Vultur gryphus*) according to age and sex, that determine colour signalling and dominance hierarchies in the carcasses. We compared the carotenoid profile in wild condors with that of captive condors fed with a controlled diet of flesh to test the hypothesis that wild individuals could acquire extra carotenoids from vegetal matter contained in carcass viscera and fresh vegetation. Wild American black vultures (*Coragyps atratus*) were also sampled to evaluate the potential influence of colouration in the integument on absorption and accumulation patterns of plasma carotenoids. A remarkably higher concentration of lutein than β-carotene was found in wild condors, while the contrary pattern was recorded in American black vultures and captive condors. We found a consistent decrease in all plasma carotenoids with age, and a lower concentration of most xanthophylls in male compared to female wild condors. Positive correlations of all carotenoids indicated general common absorption and accumulation strategies or a single dietary source containing all pigments found in plasma. The comparatively low total concentration of carotenoids, and especially of lutein rather than β-carotene, found in captive condors fed with a diet restricted to flesh supports the hypothesis that Andean condors can efficiently acquire carotenoids from vegetal matter in the wild. Andean condors seem to be physiologically more competent in the uptake or accumulation of xanthophylls than American black vultures, which agrees with the use of colour-signalling strategies in sexual and competitive contexts in the Andean condor. This study suggests that vultures may use dietary vegetal supplements that provide pigments and micronutrients that are scarce or missing in carrion.

## Introduction

Carotenoids play important roles in the production of coloured signalling traits and health maintenance by acting as provitamin A, immunomodulators, antioxidants and photoprotectants [1], [2], [3]. Animals obtain carotenoids from the food-chain as these pigments are only synthesized by primary producers [4]. The intake of carotenoids should therefore be related to their availability, accessibility and concentration in the diverse array of food consumed by animals [4], [5], [6], [7], [8]. It has been proposed that these pigments are limiting resources in the attainment of colouration due to general environmental constraints and particularly the low availability and accessibility of carotenoids in food [3]. This potential limitation can promote trade-offs in the maintenance of honest condition-dependent colouration [9], [10], although the production of carotenoid-dependent ornamentation could also be linked to the biochemical efficiency of vital cellular processes [3]. Whatever the ultimate cause or mechanisms of carotenoid allocation, the uptake of these pigments should be proximately determined by the degree to which carotenoids are limiting and by the individuals’ ability to obtain them from their diet [5], [11], [12].

Little is known about dietary carotenoid requirements for the maintenance of vital cellular processes and signalling in wild animals, or about the biochemical mechanisms that regulate these relationships via potentially shared pathways [3], [13]. Understanding these relationships requires a detailed knowledge of the dietary sources of particular carotenoids with different functions, and of their likely contrasting availability among and within habitats based on concentrations in primary producers [11], [14], [15]. The influence of these factors on the uptake and allocation of carotenoids could be modulated by foraging strategies or particular behavioural mechanisms aimed at obtaining variable amounts of each required carotenoid [5], but the mechanisms linking physiological needs to foraging behaviour are poorly understood. For instance, some species may exploit unusual foraging strategies and food sources to obtain carotenoids that are typically scarce in their diet (e.g., [11], [16]). This is suggestive of a major but neglected relevance of the proximate mechanisms used by animals to seek and choose food in order to obtain carotenoids [5], [17].

In birds, circulating carotenoid concentration is related to diet, size, colouration, phylogeny and species-specific physiological adaptations for carotenoid uptake and utilization [6], [18]. Due to the combination of large size, scarce carotenoid colouration and a diet based on rotten flesh and bones, vultures have shown the lowest concentration of plasma carotenoids amongst birds [6]. There is, however, limited information about the identity of the different circulating carotenoids and their particular concentrations in blood and bare parts in most raptors, vultures in particular. In addition to carotenoid intake, raptors often differ in integument colour and circulating carotenoid concentrations even when fed a constant diet, indicating physiological regulation by other factors such as age, sex, season, hormone levels, health status, body condition, etc. [19], [20], [21], [22]. These factors could also play a role in the differential uptake of each carotenoid type depending on their availability and accessibility in food and on their potentially different physiological functions, which remain generally unknown.

In this study, we determined the concentration of each carotenoid type present in plasma of wild Andean condors (*Vultur gryphus*) according to age and sex. These factors may influence the regulation of plasma carotenoids because Andean condors differ in presumably carotenoid-dependent colouration used in intraspecific age/sex dominance relationships at the carcasses [23]. The concentration of plasma carotenoids was also investigated in monomorphic American black vultures (*Coragyps atratus*), captured and sampled at the same time and location, feeding on the same carcasses, in order to evaluate the potential influence of colouration in the integument (lacking in the American black vulture) on absorption and accumulation patterns of plasma carotenoids. Both species feeds on carcasses of medium to large-sized vertebrates, especially of domestic ungulates in variable states of decomposition [24], [25]. Meat of domestic ungulates and other herbivore mammals has a low concentration of carotenoids [4], [26], which should degrade quickly in carrion under natural environmental conditions due to oxidation and photodegradation. Carotenoid concentration can vary among tissues in several livestock herbivores [26], [27], and thus scavengers should ingest variable, but always low, levels of these pigments depending on the tissue and the degree of autolysis [5], [11].

New and Old World vultures have been recorded consuming vegetal content of carcass viscera and fresh vegetation [28], [29], [30], but no information exists on whether these materials can be sources of dietary carotenoids for vultures. Remarkably, Egyptian vultures (*Neophron percnopterus*) show yellow facial coloration mostly dependent on lutein partially obtained by ingesting herbivore excrement rich in carotenoids [11]. Here we hypothesized that, given the scarcity of carotenoids in carrion, vultures could acquire an extra amount of these pigments by ingesting fresh vegetation and semi-digested vegetal matter contained in carcass viscera. To evaluate this hypothesis, we determined plasma concentrations of carotenoids in captive Andean condors fed with a constant diet of flesh from laboratory and livestock mammals. This determination allowed us to set a plasma carotenoid baseline concentration to assess the potential influence of the ingestion of digestive viscera and its vegetal content from entire herbivore carcasses, as a potentially rich source of carotenoids [26], [27]. We paid special attention to the concentration of each type of plasma carotenoid and its covariation within individuals in order to evaluate their potential sources and the preferential or passive physiological strategies for uptake and accumulation of dietary and metabolized pigments with different physiological functions [7]. If Andean condors are able to obtain carotenoids from vegetal matter, we predict that wild individuals should have higher plasma concentrations of these pigments (especially xanthophylls comparatively scarce in flesh) than healthy captive individuals in which β-carotene should dominate when fed on a controlled diet of flesh without viscera or fresh vegetation. Because of the lack of carotenoid-dependent colouration in the integument of American black vultures, we also predict that Andean condors should be more efficient in the uptake and accumulation of carotenoids than American black vultures feeding on the same carcasses in the wild.

## Materials and Methods

### Studied Species

**Figure 1:**
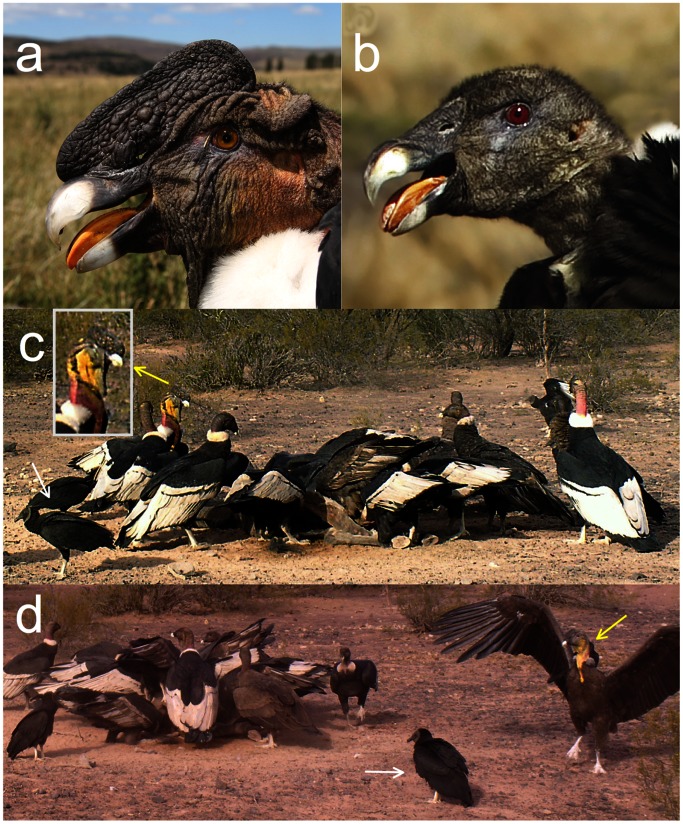
Pictures of wild Andean condors. (a) male and (b) female adult wild Andean condors showing similar orange tongue colour and different iris colour. (c) A dominant adult male Andean condor is identified by its characteristic yellow neck (enlarged picture) in a typical mix of condors and American black vultures (white arrows) gathered around a carcass. (d) A subadult male condor can be identified on the right (yellow arrow) by its less intense but still conspicuous yellow colour in the neck. Photos, a: U. Mellone, b: G. Ignazi, and c-d: V. Cailly Arnulphi.

The Andean condor is the world’s heaviest soaring bird and one of the most sexually-dimorphic bird species: adult males (∼15 kg) weigh about 30% more than females (∼11 kg). This species is the unique exception to the reversed sexual dimorphism found in raptors. The Andean condor is a globally threatened scavenger that lives in hilly areas throughout the South American Andes region. Condors are social birds aggregating at carcasses and roosting sites, where they segregate by age and sex, with adult males being at the top of the hierarchy and juvenile females at the lower rank [23], [31]. The species’ appearance differs between ages and sexes according to plumage features, morphology, colour and size of bare parts, especially in the head and neck [28], [32]. Males have a large comb, brown iris, neck wattles and bare skin varying from grey to yellow in the head and neck; females have a red iris, less yellow-coloured skin and no comb, and juveniles show dark grey bare skin. The intensity of colour in the bare parts of adults and subadults can change from pale pink, yellow or grey to deep yellow, orange and red within a few seconds during contests at carcasses, especially in males (Fig. 1), which suggests a role as dominance indicators facilitating the establishment of feeding hierarchies.

The American black vulture (∼1.5 kg) is deep black and grey in colour, except for the white wing patch and brown iris, and shows no patent external differences between ages or sexes. This species inhabits a large range throughout the Americas and shows an increasing population trend primarily due to the opportunistic exploitation of food resources associated with human activities [30].

### Sample Collection

Vultures were captured using cannon nets in October-November 2010 near Bariloche, Rio Negro, Argentinean Patagonia. A sample of blood (2–3 mL) was extracted from the brachial vein of Andean condors (n = 22) and American black vultures (n = 17), transferred to vials containing dry heparin and transported in a cooler. On the day of collection, blood samples were centrifuged at 13 000 g for 10 min to obtain plasma, which was frozen at −20°C until analysis. In addition, blood samples of captive Andean condors (n = 27) were obtained from the Buenos Aires Zoo, Argentina, where condors were fed on flesh without vegetal content from viscera: fresh muscular tissue with bones and skin from laboratory animals and herbivore livestock mammals, mostly cattle and goat. Sampled individuals included juveniles in their first, second and third calendar year, subadults (3–5 years old) and adults with definitive plumage (≥6 years old) of both sexes. Permissions to capture and sampling blood from Andean condors and American black vultures were provided by Dirección de Fauna Silvestre de Río Negro, the Argentine National Park Administration, the Buenos Aires Zoo, Argentine, and the owners and managers of local farms, who also approved the capture and sampling protocols. Age and sex determination of Andean condors were conducted according to plumage features, morphology and size [28], [32].

### Determination and Quantification of Plasma Carotenoids

An optimized procedure was used for carotenoid analysis. Briefly, a known volume of plasma (100 µL) was lyophilised, and the carotenoid pigments were extracted from the dry residue with 200 µL of N,N-dimethylformamide for 60 min, including sonication for 5 min every 30 min. The resulting extract was subsequently centrifuged at 12000 g for 5 min and the upper layer stored at −30°C until analysis by high performance liquid chromatography (HPLC). The chromatographic analysis was carried out on the same day as the preparation of the extracts. Analyses were carried out in duplicate. All operations were carried out under dimmed light to prevent isomerization and photodegradation of carotenoids.

Identification of carotenoids present in plasma samples was carried out following standard procedures [33], and consisted of the separation and isolation of the pigments by thin layer chromatography (TLC) and co-chromatography with standards, acquisition of UV-visible spectra in different solvents, as well as chemical derivatization microscale tests for the examination of 5,6-epoxide, hydroxyl and carbonyl groups [34]. The chromatographic, spectroscopic and chemical properties of the pigments were compared with authentic carotenoid samples as well as with the data in the literature [35], [36], [37], [38], [39]. Authentic pigment samples of carotenoids were isolated, and purified by means of TLC, from natural sources: β-carotene (β,β-carotene), β-cryptoxanthin (β,β-caroten-3-ol), and zeaxanthin (β,β-carotene-3,3′-diol) were obtained from red pepper (*Capsicum annuum* L.), and lutein (β,ε-carotene-3,3′-diol) from mint leaves (*Mentha arvensis*) [40]. Echinenone standard was generously given by Dr George Britton (School of Biological Sciences, University of Liverpool, UK). The identification of *cis* isomers of lutein and zeaxanthin was based on the presence and relative intensity (%A_B_/A_II_) of the *cis* peak at about 330–340 nm in UV-visible spectrum, a reduction in the fine structure and a small hypsochromic shift in λ_max_ with respect to the all-*trans* lutein, and the chromatographic behaviour in the C18 reversed phase HPLC column (the *cis* isomers are slightly more retained than the all-*trans* isomer) [38].

Quantitative analysis of carotenoids by HPLC was carried out according to the method of Mínguez-Mosquera and Hornero-Méndez [40] with some modifications. The HPLC system consisted of a Waters 2695 Alliance chromatograph fitted with a Waters 2998 photodiode array detector, and controlled with Empower2 software (Waters Cromatografía, S.A., Barcelona, Spain). A C18 reversed phase analytical column (Mediterranea SEA18, 200×4.6 mm i.d., 3 µm; Teknokroma, Barcelona, Spain) was used. Separation was achieved by a binary-gradient elution using an initial composition of 75% acetone and 25% deionised water, which was increased linearly to 95% acetone in 10 min, then raised to 100% in 2 min, and held constant for 10 min. Initial conditions were reached in 5 min. An injection volume of 20 µL and a flow rate of 1 mL/min were used. Detection was performed at 450 nm, and the online spectra were acquired in the 350–700 nm wavelength range. Quantification was carried out using external standard calibration curves prepared with zeaxanthin, lutein, β-cryptoxanthin, echinenone and β-carotene standards. Calibration curves were prepared in the range of 0.5–45.0 µg/mL, and constructed by plotting the peak area at 450 nm versus the pigment concentration. The calibration curves of all-*trans*-lutein and all-*trans*-zeaxanthin were also used to determine the concentration of their respective *cis* isomers. α-Cryptoxanthin was quantified by using the β-cryptoxanthin curve.

### Statistical Analyses

Univariate differences in the concentration of plasma carotenoids between species (wild Andean condors and American black vultures) and within species (captive and wild Andean condors) were evaluated according to the effect size of differences (Cohen’s d and their 95% confidence intervals) and t-tests on transformed data to attain normality. To determine the magnitude of effect size that constitutes a biologically relevant difference, we assessed whether lower and upper 95% confidence intervals overlapped with the zero Cohen’s d reference value indicating no difference between groups [41], [42].

Relationships between the concentrations of each type of plasma carotenoid within individuals were calculated by means of Pearson correlations. To attempt to objectively reduce the original database to smaller, mutually-uncorrelated composite variables or factors, we conducted factor analysis using the correlation matrix. Scores from the underlying factors that cause the potential covariation between observed concentrations of each carotenoid were extracted and used as independent (orthogonal) response variables.

Differences in total plasma carotenoid concentration (µg/mL) with age, sex, and status (wild or captive) of Andean condors were evaluated by means of three-way ANOVA. The variation in concentration of each type of carotenoid according to age and sex of wild Andean condors was assessed by two-way MANOVA. The models were reduced to their simplest form by eliminating, in a backward stepwise manner, any independent variables or interactions that failed to explain significant variation in the dependent variable. We proceeded by testing the significance of all variables and two-way interactions, removing only those that resulted in a lower association with the dependent variable, starting from two-way interactions. The significance of the remaining variables was tested again, until no additional variable or interactions could be removed from the model. The result is the most adequate model for explaining the variability in the response variable, where only the significant explanatory variables are retained. Full models were also reported including non-significant fixed effects terms, but excluding non-significant interactions. Partial Eta squared (*η_p_* ^2^) was used as a measure of effect size in ANOVA [41].

## Results

### Carotenoid Profile and Concentration

**Figure 2:**
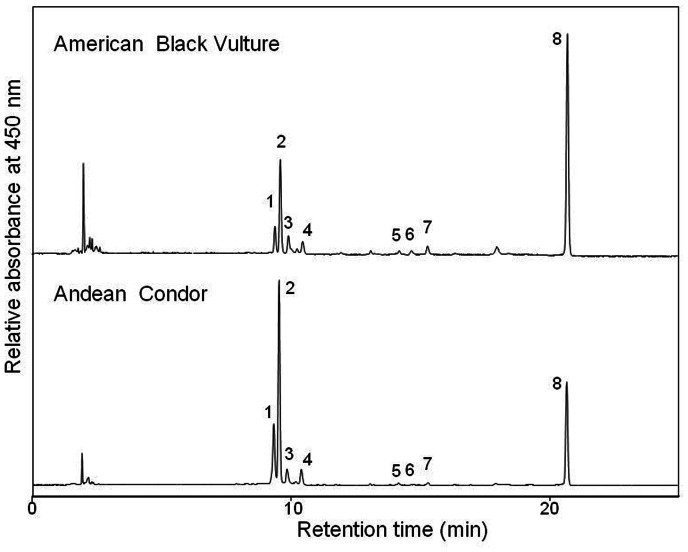
HPLC chromatograms of plasma carotenoids. C18 reversed-phase HPLC chromatogram obtained for a carotenoid extract from wild Andean condor and wild American black vulture plasma samples. Peak identities: 1, all-*trans*-zeaxanthin; 2, all-*trans*-lutein; 3 & 4, mixture of *cis* isomers of zeaxanthin and lutein; 5, all-*trans*-α-cryptoxanthin; 6, all-*trans*-β-cryptoxanthin; 7, echinenone; 8, all-*trans*-β-carotene. Detection wavelength was 450 nm.

We found carotenoid pigments in plasma of all sampled wild Andean condors (n = 22) and American black vultures (n = 17), and in most (89%) captive Andean condors (only in three of 27 individuals we do not found carotenoids). Carotenoids identified included xanthophylls (*trans*-lutein, *trans*-zeaxanthin, *cis-*lutein and *cis-*zeaxanthin isomers, *trans*-α-cryptoxanthin, *trans*-β-cryptoxanthin and echinenone) and β-carotene. The carotenoid profiles were qualitatively similar in Andean condors and American black vultures captured in the same area and on the same dates (Fig. 2), as well as in captive Andean condors (Table 1). Traces of *trans*-α-cryptoxanthin, *trans*-β-cryptoxanthin and echinenone were found in both wild and captive condors and in wild American black vultures (see Fig. 2), but not in all individuals.

**Table 1:** Carotenoid concentration (mean ± SD µg/mL) of each pigment and total carotenoid concentration in plasma of wild and captive Andean condors and in wild American black vultures.

	Andean condor		American black vulture	Effect size, Cohen’s d (95% CI)	
Pigment	captive, n = 27	wild, n = 22	wild, n = 17	captive *vs*. wildAndean condors	wild Andean condor *vs*.black vulture
Zeaxanthin	0.027±0.055	1.060±0.467	0.162±0.086	5.27 (4.02–6.36)	3.48 (2.42–4.40)
Lutein	0.064±0.154	2.210±1.110	0.460±0.244	4.56 (3.43–5.53)	2.65 (1.74–3.45)
*cis*-lutein/*cis*-zeaxanthin	0.026±0.064	0.648±0.318	0.221±0.127	4.72 (3.57–5.72)	2.31 (1.46–3.07)
α-Cryptoxanthin	0.001±0.004	0.025±0.013	0.016±0.016	3.72 (2.74–4.58)	0.86 (0.18–1.50)
β-Cryptoxanthin	0.002±0.004	0.028±0.015	0.018±0.013	3.45 (2.52–4.27)	0.74 (0.07–1.38)
Echinenone	0.009±0.012	0.037±0.017	0.044±0.021	2.10 (1.37–2.76)	−0.22 (−0.85–0.42)
β-Carotene	0.174±0.208	1.188±0.529	1.066±0.482	3.12 (2.24–3.89)	0.29 (−0.35–0.92)
Carotenoids (total)	0.304±0.453	5.197±2.060	1.987±0.844	4.54 (3.42–5.52)	2.11 (1.29–2.85)

Effect size estimations (Cohen’s d ±95% confidence intervals CI) for univariate differences in concentrations of plasma carotenoids between captive and wild Andean condors, and between wild Andean condors and wild American black vultures are shown. All comparisons between captive and wild Andean condors were statistically significant (t-test, all P<0.0001). All comparisons between wild Andean condors and wild American black vultures were statistically significant (t-test, all P<0.028), except those involving echinenone and β-carotene (t-test, both P>0.37).

**Figure 3:**
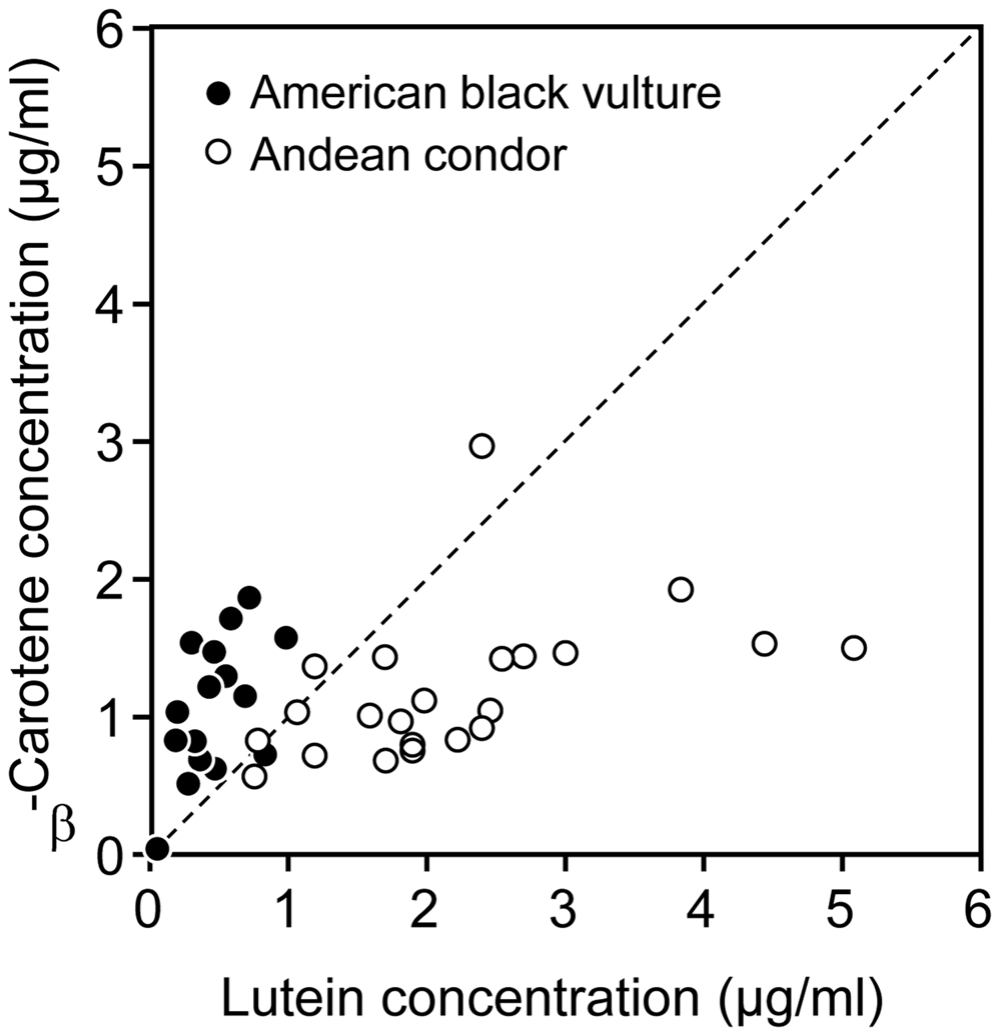
Relationships between plasma lutein and β-carotene. Relationships between concentration (µg/mL) of lutein and β-carotene in plasma of wild American black vultures (black symbols) and wild Andean condors (white symbols). Isoline is shown indicating an equal ratio between carotenoids.

The concentration of plasma carotenoids of Andean condors primarily consisted of xanthophylls (43%, especially lutein) and β-carotene (23%, Table 1). Plasma of captive condors contained the same types of carotenoids, but at much lower average concentrations (Table 1). Wild condors showed much higher mean plasma concentration of lutein, zeaxanthin, and their *cis* isomers, but similar concentrations of β-carotene than American black vultures, thus resulting in a total carotenoid concentration about three times higher in condors than black vultures; the pigments found at trace concentrations did not differ between species (univariate comparisons, Table 1). This primary between-species difference in the mean total concentration of xanthophylls rather than carotenes was due to the contrasting concentration of these pigments within individuals. Thus, plasma lutein concentration was higher than that of β-carotene within most condors, while β-carotene concentration was higher than lutein in most black vultures (Fig. 3).

### Correlations of Carotenoid Concentrations

The concentrations of all plasma carotenoid types were positively correlated within individuals, both in wild and captive condors, and especially between xanthophyll types (Table S1). Only two correlations among carotenoids from wild condors did not reach statistical significance (both involving echinenone correlations with other xanthophylls, r ≤0.39, P≥0.075, n = 22), while all relationships were positive and significant in captive condors (all r ≥0.54, all P<0.006, n = 24; three captive individuals in which we did not detect plasma carotenoids were excluded from the analysis). Similar strong and positive relationships were found when ages and sexes were analyzed separately (results not shown). Black vultures showed similar strong and positive covariation of carotenoid concentrations within individuals; 19 of 21 correlations reached statistical significance (all r ≥0.525, all P≤0.030, n = 17), especially among xanthophylls (all r ≥0.738, all P≤0.001, n = 17). The only two non-significant correlations involved echinenone relationships with other xanthophylls (both r ≤0.328, P≥0.075, n = 17).

Factor analysis using the correlation matrix among carotenoids resulted in single (captive condors) or two major axes (wild condors and black vultures) with eigenvalues greater than 1 that accounted for most of the variance (Table 2). The first factor consistently explained more than 70% of the variance and revealed high loading for xanthophylls in all cases; the second axis explained much less variance (about 15%) and revealed high loading for β-carotene and echinenone (Table 2).

**Table 2:** Factor analysis loadings for the main factors (eigenvalues >1) extracted from the correlation matrix of concentrations of each type of plasma carotenoids within individual wild and captive Andean condors and wild American black vultures.

	Andean condor			American black vulture	
	wild		captive		
Pigment	Factor 1	Factor 2	Factor 1	Factor 1	Factor 2
Zeaxanthin	0.905	0.173	0.942	0.954	0.163
Lutein	0.943	0.230	0.961	0.932	0.296
*cis*-lutein/*cis*-zeaxanthin	0.891	0.410	0.902	0.865	0.422
α-Cryptoxanthin	0.610	0.549	0.897	0.868	0.252
β-Cryptoxanthin	0.747	0.575	0.865	0.725	0.538
Echinenone	0.180	0.967	0.812	0.170	0.958
β-Carotene	0.352	0.895	0.835	0.400	0.863
Eigenvalue	5.153	1.053	5.534	5.243	1.061
% of explained variance	73.620	15.048	79.051	74.900	15.158
% of cumulative variance	88.668		79.051	90.058	

### Age and Sex Differences

**Figure 4:**
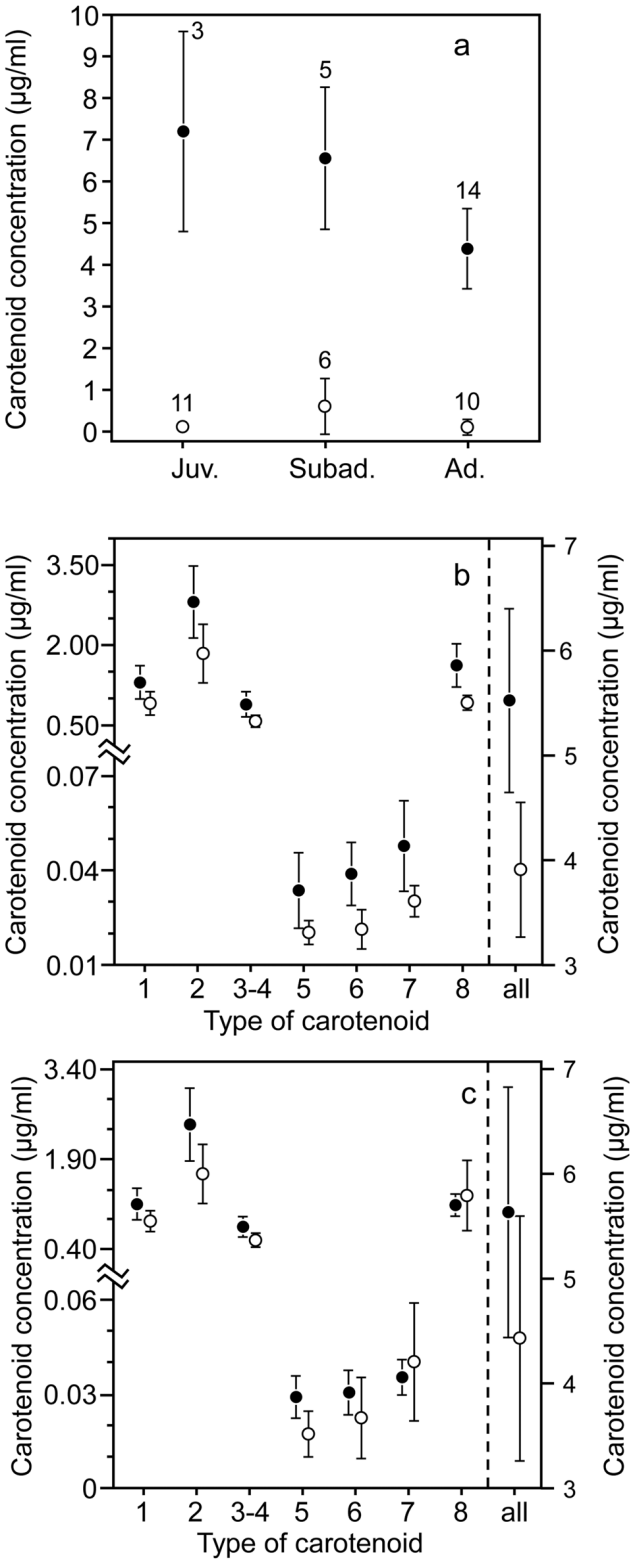
Descriptive statistics. (a) Total plasma carotenoid concentration according to age of wild (black points) and captive (white points) Andean condors; sample size is shown above bars. (b) Plasma concentration of each carotenoid type (see Fig.2 for reference) and total carotenoid concentration (right axis) according to age (pooled juveniles and subadults: black points, n = 8, adults: white points, n = 14) and (c) sex (males: white points, n = 7, females: black points, n = 15) of wild Andean condors. Points are mean values, and bars are 95% CI.

The total plasma carotenoid concentration was greater in wild condors than in captive condors (Table 3: model a, Fig.4a). In addition, total carotenoid concentration decreased with age in wild condors (Fig. 4a), but there was no apparent variation with age in captive individuals, as indicated by the interaction of both factors (Table 3: model a, Fig. 4a). There was no effect of sex or other interactions between the factors (Table 3: model a). Similar results were obtained when the three captive individuals in which we found no plasma carotenoids were excluded from the analysis.

**Table 3:** Summary of results from the ANOVA.

Dependent variable	Source	Estimate ± SE	df	F	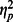	P	Adjusted R^2^
(a) Total plasma carotenoid concentration (captive, n = 27, and wild Andean condors, n = 22)							
Total concentration							
	Status	(wild) 1.536±0.149	1	276.158	0.865	<0.0001	0.868
	Age	(juv.) −0.242±0.158	2	2.305	0.097	0.112	
		(subad.) 0.056±0.186					
	Sex	(male) −0.111±0.118	1	0.883	0.021	0.353	
	Status x age	(wild x juv.) 0.849±0.278	2	4.902	0.186	0.012	
		(wild x subad.) 0.422±0.265					
(b) Particular plasma carotenoid concentration (wild Andean condors, n = 22)							
Zeaxanthin	Age	0.203±0.085	1	5.742	0.232	0.027	0.217
	Sex	−0.146±0.088	1	2.759	0.127	0.113	
Lutein	Age	0.359±0.134	1	7.189	0.274	0.015	0.299
	Sex	−0.302±0.138	1	4.768	0.201	0.042	
*cis*-Lut./*cis*-Zea.	Age	0.190±0.062	1	9.496	0.333	0.006	0.354
	Sex	−0.145±0.064	1	5.164	0.214	0.035	
α-Cryptoxanthin	Age	0.041±0.014	1	9.176	0.326	0.007	0.414
	Sex	−0.042±0.014	1	9.198	0.326	0.007	
β-Cryptoxanthin	Age	0.056±0.016	1	11.514	0.377	0.003	0.367
	Sex	−0.033±0.017	1	3.780	0.166	0.067	
Echinenone	Age	0.042±0.015	1	7.928	0.294	0.011	0.229
	Sex	0.004±0.016	1	0.083	0.004	0.776	
β-Carotene	Age	0.306±0.075	1	16.591	0.466	0.001	0.416
	Sex	0.018±0.077	1	0.057	0.003	0.814	
(c) Factor scores of major carotenoid factors (wild Andean condors, n = 22)							
Factor 1(Xanthophylls)	Age	0.791±0.373	1	4.503	0.192	0.047	0.299
	Sex	−1.048±0.385	1	7.410	0.281	0.014	
Factor 2 (Carotenes)	Age	1.102±0.381	1	8.350	0.295	0.009	0.259
	Sex	0.239±0.402	1	0.354	0.018	0.559	

Explaining (a) total plasma carotenoid concentration (µg/mL) in Andean condors according to status (wild or captive), age (juveniles, subadults and adults) and sex. (b) Models explaining variation in the plasma concentration of each carotenoid type according to age (pooling juveniles and subadults) and sex of wild Andean condors. (c) Models explaining variation in factor scores of the main carotenoid axis from a factor analysis according to age and sex of wild Andean condors. The estimated effect and standard error as well as F-values, associated probabilities and Partial Eta squared (*η* ^2^ *_p_*) are shown for those variables that significantly improved the fit of the models (final model). For those terms that were excluded from the model during the backwards procedure, F-values, P-values and *η* ^2^ *_p_* when added to the final model are given. Estimates refer to juveniles and subadults (pooled) and male wild Andean condors in models b and c.

The analysis of the plasma concentration of each particular pigment showed a consistent decrease in all pigments with age (Table 3: models b, Fig. 4b) and a lower concentration of most xanthophylls in male compared to female wild condors (Table 2: models b, Fig. 4c). In these analyses we pooled juvenile and subadult categories given the small sample size of the former (n = 3 females) after confirming no significant differences in the concentration of each carotenoid between these two age categories (post-hoc Tukey test from MANOVA, all P>0.203). There was no effect of age or sex on the plasma concentration of any of the particular pigments in captive individuals (Table S2).

Because all carotenoids were correlated (see above), we used factor scores of the factor analysis defining concentration gradients of xanthophylls (Factor 1) and β-carotene and its metabolite echinenone, carotenes hereafter (Factor 2) to assess differences between ages and sexes while avoiding covariation between particular carotenoids in wild condors. The results showed that xanthophylls differed between sexes and ages (Table 3: models c), while carotenes differed between ages but not between sexes (Table 3: models c; all age x sex interactions were non-significant).

## Discussion

The carotenoid profile in plasma of predatory and scavenger raptors showed a predominance of xanthophylls (∼90%), especially lutein, and traces of other carotenoids [11], [12], [20], [43], [44]. In this study, we found an unexpectedly large variety of carotenoids in plasma of Andean condors and American black vultures, two scavengers that mostly feed on herbivore mammal carcasses in varying states of decomposition [24], [25], [30]. Recorded plasma carotenoids can be considered dietary rather than metabolically derived, as they can be found at low concentrations in fresh tissues of domestic mammal herbivores [4], [26], except echinenone, which should be metabolically derived from β-carotene [45], [46]. Contrary to all predatory and scavenger raptor species investigated so far, high relative contributions of β-carotene were found in both species, at similar or even higher concentrations than those found in small-sized insectivorous, frugivorous and granivorous birds [7], [47]. High relative contributions of β-carotene may be explained by the fact that mammals, which prevail in the diet of vultures [28], [29], tend to accumulate carotenes to the detriment of xanthophylls [26], [27], [48], but this fails to explain the general lack of carotenes in plasma of other predatory and scavenger raptors (see above). Andean condors showed a carotenoid concentration about three times higher than that of wild American black vultures, despite their similar diets in the study area [24], [25]. This difference cannot be attributed to the contribution of β-carotene found at similar concentrations in either species, but to the highest plasma concentrations of xanthophylls in the Andean condor.

Between-species differences in plasma carotenoids and their deposition in the integument have been argued to be due to the existence of species-specific endogenous adaptations for its uptake, storage, distribution, elimination and use [6], [7], [49] or due to the impact of environmental agents influencing carotenoid absorption and transformation (e.g. [9], [19], [7], [50]). The red, orange and yellow colouration in the iris, tongue and bare skin suggests allocation of carotenoids to signalling in both sexual and competitive contexts in the Andean condor but not in the American black vulture. Andean condors have been reported to use flushing displays to change the colour of bare parts in these contests (Fig. 1). Flushing occurs through vasoconstriction of subcutaneous capillaries by using haemoglobin as pigment [51], [52]. Carotenoids can also be deposited in the skin of bare parts of birds [11], [16], which in combination with vasoconstriction and the use of blood haemoglobin have been shown to cause rapid changes in throat pouch colouration in the great frigatebird, *Fregata minor* [53]. A similar process could occur in Andean condors and other vulture species displaying a coloured and inflatable neck pouch (e.g., California condors *Gymnogyps californicus*). However, further research to ascertain the presence of carotenoids in bare parts is required. In addition to haemoglobin, the concentration of circulating carotenoids may also influence blood (plasma) colour in some species [54], [55]. A positive relationship between plasma hue and carotenoid concentration was not found at the interspecific level [55], probably because of contrasting between-species variation in diet-derived plasma carotenoid composition [7]. Whether the concentration and composition of circulating carotenoids could contribute to the colour of highly vascularised bare skin patches remains unknown.

Whatever the mechanism and pigments involved, the variation of plasma carotenoid concentration with age and sex in wild Andean condors indicated physiological regulation depending on these and other potentially interrelated factors [3], [19], [20], [22], [56]. The higher carotenoid levels in young compared to adult birds (xanthophylls and β-carotene) and in females compared to males (xanthophylls), did not support an inadequate intake of dietary carotenoids and trade-offs in their allocation for health versus colouration due to inexperience, poor nutritional state or low status of individuals [57]. In fact, less coloured young and female condors are subordinate in foraging hierarchies [23]. Circulating carotenoids may be differentially allocated to signalling colouration in adult males honestly reflecting their physiological state [3], thus explaining their lowest concentrations of plasma carotenoids. Alternatively, different physiology governs variable signalling mechanisms and patterns between sexes and ages [19], [20], [21], due to the high sexual dimorphism and other extreme life-history traits in the Andean condor [23], [32]. These kinds of speculations generally remain untested because of the complex interactions between physiological carotenoid availability, coloured trait requirements and allocation to other functions [3], [10]; for instance, egg formation or the intense red iris of female condors could require the allocation of larger carotenoid amounts, or different pigments, than the brown iris of males.

Because carotenoids come in the form of xanthophylls and carotenes, they could be taken up and accumulated differentially or serve different functions [3], [7], [48]. Positive correlations of all carotenoids in wild Andean condor and black vultures indicated a general “more is better” common absorption and accumulation strategy [58] or a single dietary source containing all pigments found in plasma. In addition, the grouping of xanthophylls and carotenes from the correlation matrix may directly reflect shared physiological pathways for xanthophylls [3] and the endogenous transformation of β-carotene to echinenone [45], [46]. This contrasts with the lack of β-carotene and echinenone grouping in captive condors, suggesting a limitation of carotenoid uptake in a diet restricted to flesh of mammals, with potential implications in health of captive condors involved in conservation programs. In any case, all carotenoids found in wild condors were also present in captive condors, indicating that flesh provided in captivity should contain all these pigments at very low concentrations [4], [26]. The pattern of absorption and plasma transport may differ between each type of carotenoid depending on its content in food but also on its metabolism and allocation to different functions [3], [7], [48], [59], [60]. Among the carotenoids found in Andean condors, β-carotene is the most abundant precursor of vitamin A (retinol) while xanthophylls may follow a different metabolic pathway to be potentially used as integument pigments and antioxidants [3]. Examining whether each type of carotenoid is involved in health and signalling is worthy of further investigation with implications for conservation of wild and captive Andean condors.

The large variety and concentration of carotenoids, especially lutein and β-carotene, and the strong positive covariation among them indicate a common and comparatively important dietary source other than flesh, which is very low in xanthophylls, especially for wild Andean condors. Dietary carotenoids have often been argued to be limiting for birds, thus promoting the traditional view focused on resource trade-offs between health and colouration [9], [10]. Alternatively, carotenoids may not be limiting in the diet of most birds [61]. Carotenoids should not be limiting for vultures if they are able to acquire them by consuming matter other than carrion, such as semi-digested plants contained in carcass viscera and faeces [11] or carotenoid-rich fresh vegetation. However, in environmental conditions with low primary production or particular population features of scavengers and their prey (e.g. low density), the scarce availability of recently dead herbivore carcasses might influence carotenoid acquisition from carrion, vegetal visceral content and fresh vegetation. As predicted, the use of food other than flesh to obtain carotenoids was supported by the low levels of all types of carotenoids found in captive condors without access to viscera and fresh vegetation. The ingestion of viscera and vegetation was likely the most important difference in diet between captive and wild condors. Also, could there be some component of the diet of wild condors that aided in carotenoid absorption/transportation (e.g. more lipids), or conversely some component of the captive diet that inhibits the uptake of carotenoids [62]. Further research is required to explore these possibilities. Remarkably, the average concentration of β-carotene in plasma of captive condors was three-fold higher than that of lutein, as expected from an exclusive diet of mammal flesh. On the contrary, the average concentration of β-carotene in plasma of wild condors was two-fold lower than that of lutein, which suggests a dietary source other than flesh to obtain this and other xanthophylls. Furthermore, it is striking that, as in captive condors but not in wild condors, American black vultures feeding on the same carcasses as wild condors showed an average concentration of β-carotene two-fold higher than that of lutein. This suggests that physiological and environmental constraints could prevent carotenoid uptake in the bloodstream from carrion and vegetal matter as a potential dietary supplement of vultures, thus explaining variation among individuals and species in the expression of carotenoid-based colouration [6].

Both Old and New World vultures consume carcass viscera and they have been rarely observed ingesting fresh vegetation [28], [29], although black vultures have been cited occasionally feeding upon vegetables and fruit [30]. Among Old World vultures, only Egyptian vultures regularly feed upon excrements [11]. New World vultures have been observed ingesting faeces of other animals, but it remains unknown if they were searching for undigested prey or particular nutrients other than carotenoids from vegetal remains [29], [30]. Lutein was the single major pigment found in Egyptian vultures, accounting for over 95% of the total carotenoid content in integument and plasma [11] despite the fact that other carotenoids, notably lutein and β-carotene, are available at high concentrations in livestock faeces [11, authors unpubl. data] and fresh vegetation [4], [62], [63]. This suggests that Egyptian vultures have a physiological incapacity or reduced capability to absorb β-carotene from flesh, viscera, faeces and fresh vegetation, or they may be very efficient at converting carotenoids, particularly β-carotene, into vitamin A. On the contrary, New World vultures investigated here were comparatively efficient in absorbing β-carotene from a similar diet or, alternatively, they were very inefficient in the conversion of this pigment into vitamin A, which is worthy of further investigation.

As predicted, Andean condors seem physiologically more competent in the uptake or accumulation of xanthophylls compared to American black vultures. These differences between species may be due to contrasting evolutionary history, physiology and associated colour-signalling strategies in the Andean condor [32]. Alternatively, this may be simply due to a higher consumption of carcass viscera, especially small intestines where carotenoids are absorbed, by Andean condors. Generally, condors are the first to access viscera because of their ability to open the peritoneal cavity of large herbivores and because of their interspecific dominance at the carcasses [25]. Carotenoids could also be acquired by directly consuming vegetal matter from the abomasum of herbivore carcasses and from fresh vegetation. A preliminary analysis of the content of Andean condor pellets (n = 135) collected in communal roosts indicated a high prevalence (∼90%) of vegetal remains; about a 35% of the pellets with vegetal remains were primarily constituted (80% of volume) by this matter. This supports the hypothesis of the consumption of vegetal matter as a potential dietary supplement to acquire carotenoids, both by ingesting natural fresh vegetation and vegetal content of herbivore carcass. It is also worth noting that plant mechanical homogenization by mastication and digestion by mammals has the potential to increase the bioavailability of carotenoids in the intestine [64], [65], [66], thereby enhancing the absorption by vultures consuming semi-digested vegetal content of carcasses. More research is needed on the feeding behaviour of vultures to assess their preference for specific carcass tissues and their degree of ‘herbivory’, whether aimed or not at the active or passive acquisition of particular micronutrients such as carotenoids, vitamins or minerals with a likely important but neglected role in their physiology and life-history.

## Supporting Information

Table S1 Correlations (Pearson r, P-values in brackets) among concentrations of carotenoid types within plasma of wild Andean condors (n = 22, upper right quadrant) and captive Andean condors (n = 24, lower left quadrant).
(DOC)

Table S2 Descriptive statistics and summary of results from the ANOVA explaining variation in the plasma concentration (µg/mL) of each carotenoid type according to age: adults (n = 10) and subadults (n = 17, pooling juveniles and subadults) and sex (male, n = 14, female, n = 13) of captive Andean condors.
(DOC)
