# Supplementary material for: Need and Seek for Dietary Micronutrients: Endogenous Regulation, External Signalling and Food Sources of Carotenoids in New World Vultures
Source: PLoS One. 2013 Jun 13;8(6):e65562. doi: 10.1371/journal.pone.0065562 (PMC3681859; doi:10.1371/journal.pone.0065562)
Supplement: Table S1 — Correlations (Pearson r, P-values in brackets) among concentrations of carotenoid types within plasma of wild Andean condors (n = 22, upper right quadrant) and captive Andean condors (n = 24, lower left quadrant). (DOC) [file pone.0065562.s001.doc]

Table S1.

|  | Zeaxanthin | Lutein | *cis*-Lut./cis-Zea. | α-Cryptoxanthin | β-Cryptoxanthin | Echinenone | β-Carotene | Total |
| --- | --- | --- | --- | --- | --- | --- | --- | --- |
| Zeaxanthin |  | 0.846  (<0.0001) | 0.835  (<0.0001) | 0.600  (0.003) | 0.716  (<0.0001) | 0.366  (0.094) | 0.503  (0.017) | 0.884  (<0.0001) |
| Lutein | 0.985  (<0.0001) |  | 0.935  (<0.0001) | 0.642  (0.001) | 0.858  (<0.0001) | 0.387 (0.075) | 0.559  (0.007) | 0.958  (<0.0001) |
| *cis*-Lut./*cis*-Zea. | 0.889  (<0.0001) | 0.909  (<0.0001) |  | 0.780  (<0.0001) | 0.906  (<0.0001) | 0.553  (0.008) | 0.666 (0.001) | 0.961  (<0.0001) |
| α-Cryptoxanthin | 0.789  (<0.0001) | 0.832  (<0.0001) | 0.782  (<0.0001) |  | 0.681  (<0.0001) | 0.633  (0.002) | 0.592  (0.004) | 0.716  (<0.0001) |
| β-Cryptoxanthin | 0.764  (<0.0001) | 0.779  (<0.0001) | 0.696  (<0.0001) | 0.971  (<0.0001) |  | 0.664  (0.001) | 0.793  (<0.0001) | 0.920  (<0.0001) |
| Echinenone | 0.644  (0.001) | 0.669  (<0.0001) | 0.632  (0.001) | 0.563  (0.004) | 0.541  (0.006) |  | 0.911  (<0.0001) | 0.597  (0.003) |
| β-Carotene | 0.671  (<0.0001) | 0.705  (<0.0001) | 0.648  (0.001) | 0.634  (0.001) | 0.595  (0.002) | 0.926  (<0.0001) |  | 0.747  (<0.0001) |
| Total | 0.889  (<0.0001) | 0.913  (<0.0001) | 0.850  (<0.0001) | 0.772  (<0.0001) | 0.720  (<0.0001) | 0.871  (<0.0001) | 0.930  (<0.0001) |  |
