# Supplementary material for: Need and Seek for Dietary Micronutrients: Endogenous Regulation, External Signalling and Food Sources of Carotenoids in New World Vultures
Source: PLoS One. 2013 Jun 13;8(6):e65562. doi: 10.1371/journal.pone.0065562 (PMC3681859; doi:10.1371/journal.pone.0065562)
Supplement: Table S2 — Descriptive statistics and summary of results from the ANOVA explaining variation in the plasma concentration (µg/mL) of each carotenoid type according to age: adults (n = 10) and subadults (n = 17, pooling juveniles and subadults) and sex (male, n = 14, female, n = 13) of captive Andean condors. (DOC) [file pone.0065562.s002.doc]

| Dependent variable | Source | Mean ± SD | df | F | P | $\eta_{p}^{2}$ | Adjusted R^2^ |
| --- | --- | --- | --- | --- | --- | --- | --- |
| Zeaxanthin | Age | subad.= 0.024±0.061, ad.= 0.032±0.045 | 1 | 0.006 | 0.937 | 0.000 | 0.022 |
|  | Sex | male=0.043±0.072, female=0.009±0.015 | 1 | 1.991 | 0.171 | 0.077 |  |
| Lutein | Age | subad.= 0.066 ±0.186, ad.= 0.062±0.082 | 1 | 0.008 | 0.928 | 0.000 | 0.001 |
|  | Sex | male=0.102±0.205, female=0.024±0.048 | 1 | 1.703 | 0.204 | 0.066 |  |
| *cis*-Lut./*cis*-Zea. | Age | subad.= 0.028±0.076, ad.= 0.023±0.042 | 1 | 0.255 | 0.618 | 0.011 | 0.018 |
|  | Sex | male=0.040±0.084, female=0.011±0.031 | 1 | 1.545 | 0.226 | 0.060 |  |
| α-Cryptoxanthin | Age | subad.= 0.002±0.005, ad.= 0.001±0.002 | 1 | 0.229 | 0.637 | 0.009 | 0.058 |
|  | Sex | male=0.002±0.005, female=0.001±0.003 | 1 | 0.547 | 0.467 | 0.022 |  |
| β-Cryptoxanthin | Age | subad.= 0.001±0.004; ad.= 0.002±0.004 | 1 | 0.003 | 0.954 | 0.000 | 0.059 |
|  | Sex | male=0.002±0.005, female=0.001±0.003 | 1 | 0.486 | 0.492 | 0.020 |  |
| Echinenone | Age | subad.= 0.008±0.012; ad.= 0.013±0.012 | 1 | 1.301 | 0.265 | 0.051 | 0.015 |
|  | Sex | male=0.011±0.012, female=0.008±0.012 | 1 | 0.000 | 0.987 | 0.000 |  |
| β-Carotene | Age | subad.= 0.146±0.212; ad.= 0.220±0.205 | 1 | 1.069 | 0.311 | 0.043 | 0.028 |
|  | Sex | male=0.197±0.234, female=0.148±0.183 | 1 | 0.000 | 0.987 | 0.000 |  |

Table S2.
